# Grin and bear it! Neural consequences of a voluntary decision to act or inhibit action

**DOI:** 10.1007/s00221-012-3263-9

**Published:** 2012-09-25

**Authors:** Elisa Filevich, Patrick Haggard

**Affiliations:** Institute of Cognitive Neuroscience, University College London, London, UK

**Keywords:** Internal inhibition, Itch, Action inhibition, Self-control

## Abstract

Action inhibition is an important part of everyday human behaviour. Most previous studies of action inhibition have focussed on stop-signals. Here, we consider the case where participants themselves decide to inhibit, or not inhibit, a prepotent action. Participants received electric stimulation that elicited an itchy feeling on the wrist. If they made a hand withdrawal movement, this would interrupt the stimulation, and halt the itch. In a factorial design, participants were given external instructions to withdraw their hand when they felt the itch, or to inhibit the natural withdrawal response, and bear the itch. In another condition, they were asked to internally choose between withdrawal and inhibition of withdrawal. Event-related potentials revealed differences between processing of the sensory consequences of internally decided and externally–instructed action and inhibition decisions. Specifically, potentials evoked by itchy stimuli were enhanced in internally decided inhibition trials, as compared to externally instructed inhibition trials. In contrast, processing of itchy stimuli was reduced in internally decided action trials, as compared to externally instructed action trials. These results show that internal decisions lead to different perceptual processing of the consequences of action and inhibition and suggest that features of decision processes can be measured via their consequences.

## Introduction

Inhibition of action is an important part of human behaviour. Most people recognize the familiar situation of having to appeal to their self-control to overcome urges to eat, drink, buy or choose something. A common reason for this internal inhibition is our knowledge that we would regret the action in the future, however, attractive it may be in the present.

### Externally instructed inhibition

Most research on the capacity to inhibit actions has studied the neural processes triggered by an exogenous instruction to inhibit (Logan 1994; Verbruggen and Logan 2008). In these tasks, participants generally make simple prepotent actions, such as manually responding to GO signals. They occasionally and unpredictably are presented with an additional NO GO signal or STOP signal, which instructs them to inhibit the prepotent response, and withhold the instructed action. Several decades of research using these paradigms have confirmed the importance of the frontal lobes in inhibitory processing (Aron et al. 2004).

However, there is an emerging consensus on the need to study inhibition with richer and more complex tasks (Aron 2011). Brass and Haggard (2007) coined the term ‘intentional inhibition’ to refer to inhibition that arises internally, from an internal decision to withhold an action, rather than from an external signal (Brass and Haggard 2007). Here, we use the more neutral term ‘internal inhibition’. This concept partly overlaps with the concept of willpower developed in behavioural social psychology (Baumeister et al. 2007), in that both have the effect of preventing or delaying inappropriate actions.

### Inhibitory self-control

Recent experimental work has given rise to the idea that willpower, or self-control, is a general capacity, or limited resource analogous to the body’s physical energy (Hagger et al. 2010). The inhibitory mechanisms associated with willpower relate to a general state in which inhibition is continuously present, until exhausted. However, internal inhibition might also involve a temporally specific decisional process, analogous to external inhibition triggered by a stop signal. For example, it has been argued that people may withhold an action at the last possible moment (Libet 1985). Therefore, in an event-related framework for internal inhibition, inhibition should appear not only as a general, sustained mental process, but also as a specific, clearly timed event. That is, the decision to inhibit may be taken in the context of a specific stimulus, and may have specific consequences. Treating inhibition as event-related would allow the neural mechanisms to be measured more precisely.

Experimental constraints raise significant methodological problems, notably for ecological validity. Most previous experimental studies of ‘free will’ have not given participants clear reasons for choosing to act or inhibit and thus have low ecological validity (Libet et al. 1983; Brass and Haggard 2007). Perhaps, the need for methodological simplicity has meant that decisions, urges, values and consequences of actions have been conspicuously missing in these paradigms.

### Measuring the consequences of inhibition

One recent experiment suggests that the sensory consequences of action could be useful to describe the processes of action that were in fact inhibited. Shocks delivered to participants’ fingers were perceived as weaker after action inhibition triggered by an external stop signal, as compared to a passive detection task (Walsh and Haggard 2010), suggesting that some characteristics of voluntary action are maintained even if the action itself is inhibited. An experimental framework to study internal inhibition of action requires three components. First, there must be a reason to perform an action. Second, the participant must make an internal decision to inhibit that action on some occasions. Third, there must be some way of measuring the internal processes associated with inhibition, rather than merely recording whether an action occurred or was inhibited.

### Resisting the urge to scratch an itch

Most people can recognize the urge to scratch an itch. *Not* scratching can be extremely effortful, and it can make the itchy feeling more intense. We developed an experimentally controlled version of this situation to create a paradigm that would meet these requirements.

In this ecologically valid task, reasons for actions were provided by delivering on each trial itchy and unpleasant stimuli that could be avoided by doing a hand movement. In this way, decisions to act or inhibit would have meaningful consequences. Internal decisions about action and inhibition were allowed in some trials, whereas clear external instructions were provided in other trials. Electroencephalography (EEG) was recorded to measure brain activity.

Specifically, participants were either instructed or had to decide whether to move their arm to avoid an unpleasant itchy sensation, or to inhibit the urge to move the arm, and withstand the itch. Thus, a strong motivation to act was introduced.

We compared event-related potentials (ERP) for situations in which inhibition of action followed either from an external instruction or from an internal decision. The ERP method allowed us to focus specifically on the sensory processing of the itchy stimuli themselves. Sensory processing of itchy stimuli produces a strong event-related potential (Mochizuki et al. 2008). We took advantage of the good temporal consistency of these known potentials to apply EEG techniques to compare these different processes across conditions.

Our main hypotheses were as follows: First, a decision to execute or inhibit action will influence subsequent sensory processing. Second, and most importantly, this influence will vary with the source of the decision: internal decisions would have different ‘downstream’ effects on sensory processes from externally instructed decisions.

## Materials and methods

### Participants

Sixteen naïve paid healthy volunteers (9 females, mean age 25.3 ± 5 years) participated in the experiment. Participants with sensitive skin were excluded from taking part in the study. Procedures were approved by the UCL Research Ethics Committee and were in accordance with the principles of the Declaration of Helsinki. One participant was excluded after participation due to excessive blinking.

Participants sat comfortably at a table and 60 cm away from a computer screen. Both hands rested comfortably on the table, so that their right index finger would rest on a force-sensitive resistor (FSR) (Active Robots Ltd, Somerset, UK), connected to a computer.

### Electrical stimuli

Itchy stimuli were delivered using previously established methods (Mochizuki et al. 2008) with some adaptations. Briefly, each electrode consisted of four pairs of stainless steel wire 0.1 mm in diameter. Each pair formed a cross and was placed approximately 2 mm away from the next pair. Current for each electrode was supplied by a Digitimer medical stimulator (Digitimer Ltd, Hertfordshire, England) and flowed through all wires. The reference electrodes (cathodes) were placed 1 cm laterally to each itch electrode. Current was delivered through the electrodes in square pulses of 2-ms duration at 50 Hz. A stepwise procedure was used to determine the current intensity necessary to elicit an unpleasantly itchy sensation in each participant. The current was explicitly set to a level that would rather be avoided, but would still be bearable. Participants were asked to rest their right hand on a force-sensitive pad. Electrical stimulation would flow through the electrodes on their left forearm only as long as the pad was touched by the right hand. As soon as the participant withdrew their hand from the pad, stimulation would stop and the itchy sensation would cease. Thus, participants could stop an unpleasant sensation on their *left* wrist by actively withdrawing their *right* hand from a resting position. Critically, whilst participants held their hand in place on the pad they actively inhibited the urge to act.

As expected from previous studies (Mochizuki et al. 2009), participants reported a strong habituation of the itchy sensation with repeated stimulation. Therefore, two separate itch electrodes were placed on the left wrist. Stimulation lasted for 3 s altogether and consisted of three 1-s-long shocks, alternating from one electrode to the other. The first electrode to be stimulated (proximal or distal to the wrist) alternated across trials.

### Task and experimental design

**Fig. 1:**
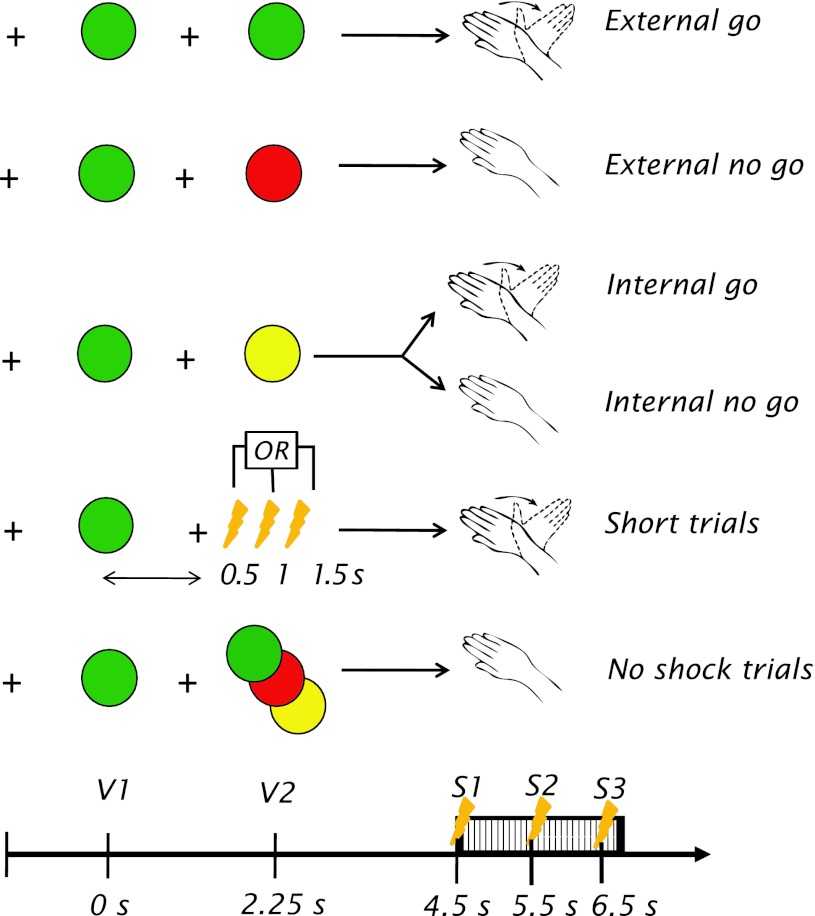
Experimental task. A visual warning sign (V1) was presented for 250 ms. After a 2-s interval, an instruction sign (V2) was presented, also for 250 ms. After a second 2-s interval, three consecutive electrical shocks were delivered at 50 Hz and for a maximum of 3 s, generating an itchy feeling on the participants’ wrist. If participants moved their hand, they would interrupt the itchy feeling. *Green-* and *red*-*coloured* V2 corresponded to external instructions to move the hand or not. *Yellow* V2 allowed the participants themselves to choose between the two possible action outcomes. Short trials in which V2 was replaced at variable times by a surprise shock were presented to encourage and probe motor preparation. Catch trials with no shock delivered were presented to ensure that participants were waiting for the shock to execute their decision

The experiment consisted of 10 blocks of 40 trials each, and lasted 80–90 min. Each trial was organized as follows: (see Fig. 1) a black fixation cross appeared over a grey background for a variable duration of between 2 and 3 s. Two visual stimuli (called V1 and V2) were presented sequentially. These signalled respectively the start of each trial, and the instructions for a given trial. V1 was a green circle subtending 1.5° at a distance of approximately 60 cm, appearing for 250 ms to mark the initiation of the trial. Participants were asked to prepare a right-hand movement as soon as V1 appeared. The fixation cross then appeared again on screen for 2 s, until a second circle appeared (V2), again for 250 ms. The luminosity of V1 and V2 was balanced with an heterochromatic flicker test with an independent set of 5 participants. This was done to adjust the intensity of the visual stimuli independently of their absolute luminance, but depending on participants’ sensitivity (Wyszecki and Stiles 1982). V2 was of the same size as V1 and could be of three different colours. If V2 was green, participants should remove their right hand as soon as they felt the shock on their left, thus terminating the shock.

The green V2 represented an ‘*external* *go’* condition, in which participants were instructed by an external instruction to perform a movement. If V2 was red, participants were asked to endure the shock and were not to move their hands. Hence, the red V2 represented the ‘*external no go*’ condition, in which participants were instructed again by an external instruction, to avoid doing any movement despite having the desire to do so.

Alternatively, V2 could be yellow, in which case the participants were asked to decide whether they would endure the shock or withdraw their hand as soon as they felt the shock, thus avoiding prolongation of the itchy sensation. In the yellow V2 condition, participants had to decide whether each trial would be a ‘*go*’ or ‘*no go*’. Hence, depending on whether the participant decided to move or not, the yellow V2 could produce an ‘*internal go*’ or ‘*internal no go*’ condition. Participants were encouraged to choose to withdraw their hand in roughly 50 % of the yellow V2 trials. After each block of 40 trials, they received feedback if the rate of withdrawal was higher than 70 % or lower than 30 % of the trials. Because participants preferred to withdraw their hands whenever they had the choice, this manipulation ensured that the overall number of trials were comparable across conditions for the EEG analysis. The block-by-block feedback and relatively loose boundaries around 50 % were included to prevent participants from developing a very strict strategy.

In this way, the experiment followed a factorial 2 × 2 experimental design, with the factors of source (internal/external) and outcome (*go*/*no go*). Critically, there was no behavioural difference between the internal and external conditions, so any differences found in the recorded neural signal associated with the internal or external sources of decision would necessarily reflect differences in the processing of internal vs. external decisions.

Two additional conditions were included for methodological reasons: 15 % ‘catch’ trials (without a shock) were presented to ensure that participants waited for the first shock before executing their internal decision or instruction and did not simply predict its onset. All three V2 colours were followed by catch trials with equal probability. In addition, to encourage movement preparation, 25 % of ‘short’ trials were included. In these trials, itch stimulation was delivered at either 0.5, 1.0 or 1.5 s after V1, in contrast with the normal time of 2,250 ms. Participants were asked to withdraw their hand from the FSR as quickly as possible in these cases.

The mean intensity at which participants reported to feel an unpleasant but bearable itchy sensation was 0.36 ± 0.14 mA at the beginning of the experiment for both electrodes. After each block, intensity was readjusted if the stimulation was perceived as too painful or too mild. Intensity never exceeded 0.4 mA, and by the end of the experiment, the mean intensity at which subjects perceived the itchy sensation was 0.38 ± 0.14 mA and 0.38 ± 0.15 mA, respectively, for each one of the stimulators.

### Electrophysiological recordings and signal analysis

A SynAmps amplifier system and Scan 4.3 software (Neuroscan, El Paso, TX) were used to record EEG data. Activity from fourteen scalp electrodes was recorded (F3, Fz, F4, FC3, FCz, FC4, C3, Cz, C4, P3, Pz, P4, O1, O2, according to the 10–20 system). The reference electrode was AFz, and the ground electrode was placed on the chin. All electrode impedances were kept below 5 KΩ. The left and right mastoids were recorded. Horizontal electrooculogram (EOG) was recorded from bipolar electrodes placed on the outer canthi of each eye, and vertical EOG was recorded from bipolar electrodes placed above and below the right eye. EEG signals were amplified and digitized at 500 Hz.

EEG data were analysed with EEGLAB software (Delorme and Makeig 2004). Data were first re-referenced to the linked mastoids. Because long epochs (8.25 s) were defined, data were digitally high-pass filtered over 0.5 Hz to remove low frequency drifts. In addition, we computed the amplitude of event-related potentials (ERPs) as peak amplitude values. A 30 Hz low-pass filter was applied to the data (Mochizuki et al. 2008). Continuous EEG data were time-locked to the trial start (stimulus V1) and baselined to the period of 250 ms to 150 ms prior to the onset of V1. To avoid artefacts due to eye blinks, trials were discarded if the bipolar recording of EOG exceeded ± 80 μV at any point during the epoch. The mean percentage of rejected trials was 22 %. This value is relatively high, but perhaps unsurprising given the long epochs and the unpleasantness of the experience. The components in the evoked response were identified by inspection of the grand-average pooling across all conditions. For each component identified in the grand average, the time of maximum amplitude of the individual average was determined and the values for each participant in that time point were computed.

## Results

### Behavioural results

Participants rarely moved their hands in catch trials (mean ± SD commission errors 0.70 ± 0.91 %).

**Fig. 2:**
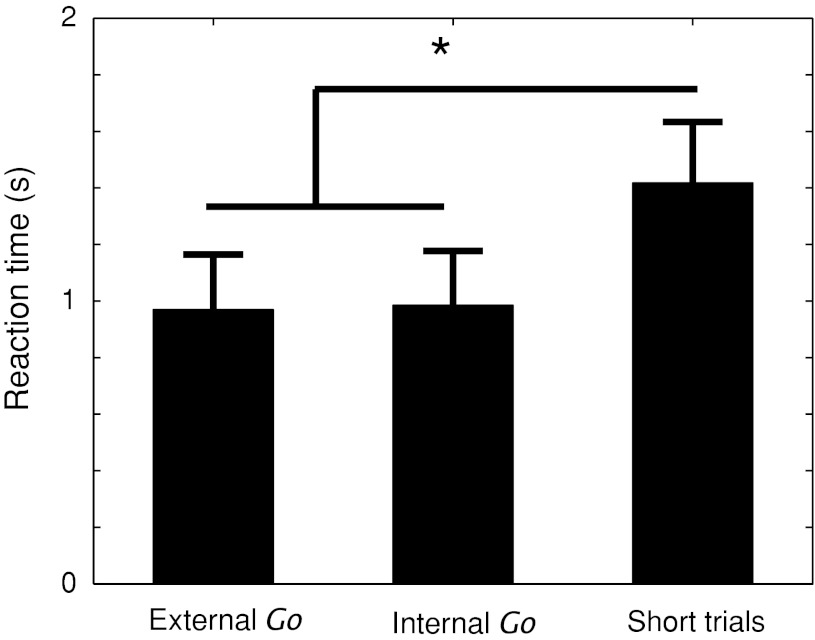
Behavioural results. Hand withdrawal times for the movement conditions. No difference is observed between the two movement conditions, in which the time of shock was highly predictable. Longer withdrawal times are observed in ‘short’ trials, suggesting that motor preparation had occurred in both internal and external *go* trials

On average, within the internal trials, participants decided to withdraw their hands (internal *go*) on 46 ± 5 % (mean ± SD) of the trials. To measure the extent of preparation of the action to withdraw the hand, we calculated the average RT to withdraw the hand after receiving an itchy shock. The RT was compared across the *internal and external* *go* conditions (for which the withdrawal movement could be anticipated and prepared) and the average of all ‘short’ trials, in which the shocks occurred without a prior V2 warning signal, therefore not allowing for movement preparation (Fig. 2).

A repeated measures ANOVA revealed a main effect of condition (*F*_1,15_ = 19.38, *p* < 0.001). RTs were longer for short trials as compared to both internal *go* and external *go* conditions. Paired comparisons revealed significant differences between the internal *go* condition and the short trials (*t*_15_ = −6.22, *p* < 0.001) and between the external *go* condition and the short trials (*t*_15_ = −5.25, *p* < 0.001). No significant differences were found between the RTs for the internal and *external go* conditions (*t*_15_ = −0.54, *p* = 0.59). These results suggest that there was movement preparation in the two *go* conditions that was less efficient in the *short* trials. It is also possible that *go* trials had shorter RTs due to the timing of the shocks becoming more predictable. Our design cannot distinguish between these two possibilities.

We observed relatively long RTs (of around 1 s). Such long reaction times may partly reflect the peculiar nature of this stimulation. An interesting feature of this itch stimulus is the lack of a discrete perceptual onset at the start of the shock-train. Short trains do not produce any sensation at all. At the intensities we used, sensory perception begins only some time after the onset of the stimulation. Because the reaction time is measured from the onset of the stimulus, the reaction time is artificially increased by delay, which we attribute to accumulation of signals in perceptual areas.

These behavioural results did not change when we excluded the participant that was excluded from the ERP analysis due to excessive blinking.

### ERP results

**Fig. 3:**
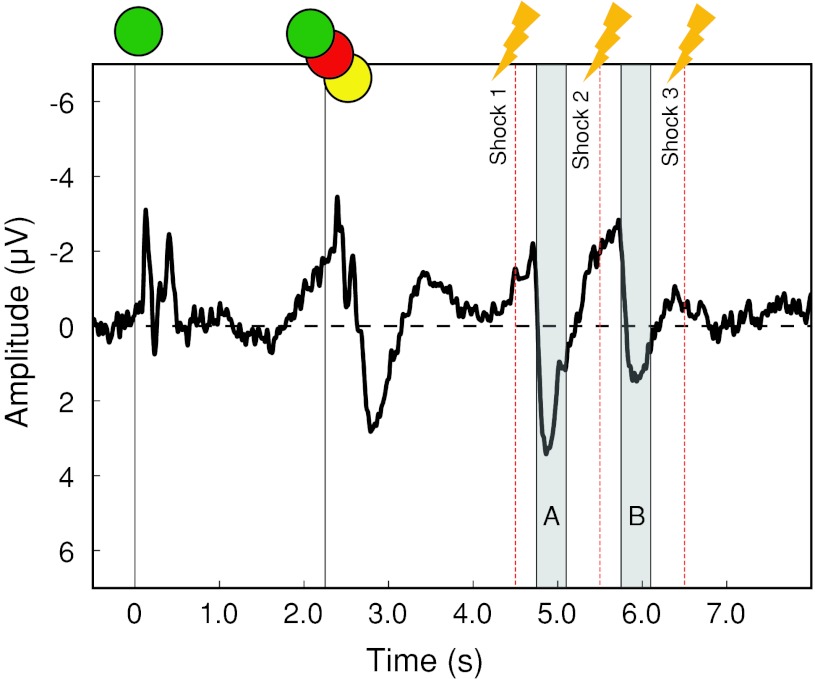
EEG results Grand average across all the four main experimental conditions (internal and external *go* and *no go*) for electrodes C3, Cz and C4. Long (8 s) epochs were visually inspected to identify two main periods of interest: response to shock 1 (*A*) and response to shock 2 (*B*). The response to shock 3 was greatly attenuated and was therefore not analysed. EEG trace is plotted negative-up

After blink rejection, an average of 42 ± 16 trials (SD) were recorded for the internal *go* condition, and 41 ± 17 trials were recorded for the internal *no go* condition. 42 ± 13 trials were recorded for the external go condition, and 49 ± 13 trials fell into the external *no go* condition. The grand-average ERPs were displayed time-locked to V1, to reveal the sequence of sensorimotor events in each epoch. Figure 3 shows the grand-average trace at C3, Cz and C4 pooled across all conditions. There is a stereotyped response to the onset of both V1 and V2. Importantly, although V1 and V2 are physically similar, only V2 carries information about the subsequent task instructions. Accordingly, the neural processing of V1 differs strongly from that of V2, with only V2 eliciting a strong positivity peaking at around 580 ms after V2 onset. There is a characteristic negativity preceding V2, recalling the CNV (Lumsden et al. 1986). This negativity starts roughly 800 ms before the onset of the first shock is visible in the grand average across conditions. Finally, the neural response to the three consecutive shocks is apparent. A marked positive-going component occurs in response to each of the three shocks, peaking at around 400 ms after shock onset.

The key ERP components evoked by the first two shocks are indicated by shaded areas *A* and *B in* Fig. 3. Figure 3 shows an average of all conditions, including both *go* and *no go* conditions. Therefore, whilst the first shock was always delivered, and acted as a GO signal, the second and third shocks were not experienced if participants withdrew their hand. In addition, the third shock was hardly perceived, even on *no go* trials, due to habituation. It was therefore not included in the analysis. Peak amplitudes for each of these events were analysed for each condition separately.

Our analysis focussed on the effects of source of decision (internal or external) and decision outcome (*go* or *no go*) on the neural activity evoked by the shocks.

Our analyses were restricted to central and parietal electrodes, because we were interested in assessing the neural consequences of inhibition over sensory processing, rather than the frontal mechanisms that cause inhibition itself (Aron et al. 2004).

### Pre-shock components

We first examined the response to the instruction cue. We chose a time window of 350–550 ms after V2. In accordance to the topographical distribution in the half-point of the chosen time window, electrodes C3, Cz, C4, P3, Pz and P4 were averaged. A 2 × 2 ANOVA of the peak amplitudes revealed a trend for a main effect of outcome (*F*_1,14_ = 3.42, *p* = 0.08), with a stronger V2 positivity in the no go conditions. There was no main effect of source (*F*_1,14_ = 0.39, *p* = 0.53) or interaction effect (*F*_1,14_ = 0.03, *p* = 0.59).

We then examined the preparatory activity the V2 instruction and before shock 1. An inspection of the grand average (Fig. 3) shows that there is an RP/CNV component before shock 1(Kornhuber and Deecke 1965; Walter et al. 1964). Topographical maps showed that this component was maximal between C3 and Cz. It was measured as the mean amplitude during the 200 ms prior to the shock for the average of these two electrodes. A 2 × 2 ANOVA of the RP/CNV amplitude revealed a main effect of outcome (*F*_1,14_ = 5.00, *p* = 0.042). This arose because preparatory negativity was significantly stronger for go trials compared with no go trials. However, there was no main effect of source (*F*_1,14_ = 2.86, *p* = 0.11), and no significant interaction between source and outcome (*F*_1,14_ = 0.57, *p* = 0.46).

### Evoked responses to the shocks

**Fig. 4:**
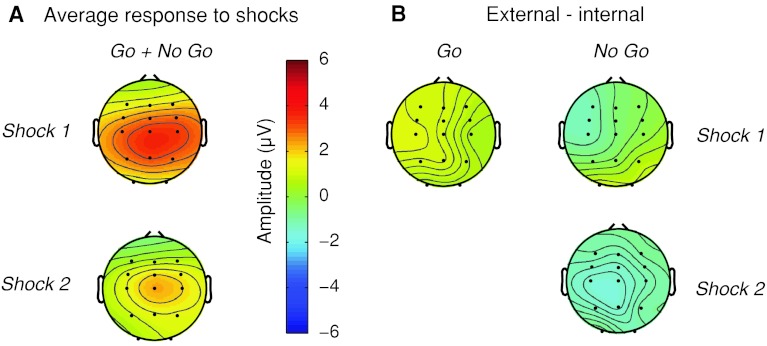
Topographical distribution of the neural responses to the shocks. The topographical maps for the response to shock 1 were calculated at 4,900 ms, and the response to shock 2 were calculated at 5,900 ms from V1 onset. **a** Average of all conditions. The topography includes an average of four conditions (external *go*, external *no go*, internal *go*, internal *no go*). Shock 2 includes external *no go* and internal *no go* conditions only. **b** Differences between external and internal conditions, for both *go* and *no go* trials

We examined the average topography of the response to shocks 1 and 2 (see Fig. 4a). As expected from previous results, the average topographical maps show that the response to the both shocks is focused on the central electrodes. Based on this topography, peak amplitudes for the analysis were obtained from the average of electrodes C3, Cz and C4.

We also examined the differences between the external and internal conditions for both *go* and *no go* trials separately (see Fig. 4b). The topographical maps show large differences in the left hemisphere, ipsilateral to the shocks but contralateral to the movement. This suggests that this difference is related to motor preparation. The most parsimonious, albeit speculative, interpretation is that go trials show a stronger (more negative) RP-like movement preparation component for internal than for external trials. This leads to a positivity observed in the electrodes contralateral to movement in the external–internal subtraction.

### Evoked response to shock 1

**Fig. 5:**
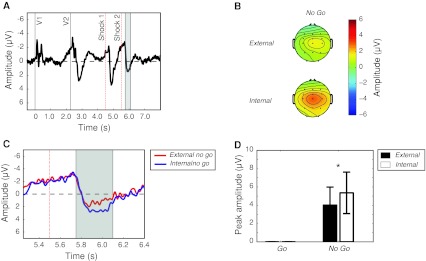
Neural response to shock 1 (**a**) Time window (4,750–5,100 ms) in which the amplitude of the response to shock 1 was measured. The* vertical dashed line* indicates the time of shock onset. **b** Scalp distribution at 4,900 ms. **c** Detail of time window of interest, showing the averaged trace for electrodes C3, Cz and C4 for each condition. **d** Mean of maximum amplitude for each subject within the selected time window, for the average of electrodes C3, Cz and C4. *Error bars* show confidence intervals. A significant interaction effect emerges (*p* < 0.01). Post hoc *t* tests show a crossover effect. Shock 1 ERPs are stronger for external *go* trials as compared to internal *go* trials (*p* < 0.05), whereas ERPs to external *no go* trials are weaker than those for internal *no go* trials (*p* < 0.05)

The topography and peak amplitude of the response to shock 1 is shown in Fig. 5 (highlighted section *A* in Fig. 3).

A 2 × 2 ANOVA of the average revealed no main effect of outcome (*F*_1,14_ = 0.06, *p* = 0.80) nor main effect of source (*F*_1,14_ = 0.01, *p* = 0.92) but a significant interaction effect (*F*_1,14_ = 19.433 *p* = 0.001), showing a crossover form in ure 5C. Post hoc *t* tests revealed that the neural response evoked by shock 1 was greater in external *go* than in internal *go* trials (*t*_14_ = 3.39, *p* = 0.004). Conversely, the response evoked by shock 1 in external *no go* trials was weaker than that evoked by the internal *no go* trials (*t*_14_ = −2.22, *p* = 0.04).

### Evoked response to shock 2

**Fig. 6:**
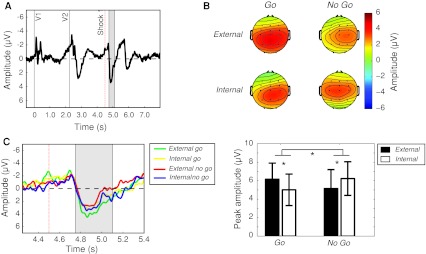
Neural response to shock 2. Because shock 2 was only fully delivered in inhibition trials, only *no go* trials are analysed. **a** Time window (5,750–6,100 ms) used to measure the amplitude of the response to shock 2. The *vertical dashed line* indicates the time of shock onset. **b** Scalp distribution at 5,900 ms. **c** Detail of time window of interest, showing the averaged trace for electrodes C3, Cz and C4 for each condition. **d** Mean of maximum amplitude for each subject within the selected time window, for the average of electrodes C3, Cz and C4.* Error bars* show confidence intervals. As in shock 1, external *no go* trials evoke a weaker response to shock 2 than an internal *no go* trials (*p* < 0.05)

Because the mean RT to withdraw the left hand was generally shorter than 1 s, shock 2 was generally not delivered in trials where participants made the withdrawal action with their right hand. Hence, the analysis of the ERP to shock 2 was confined to *no go* trials, in which participants did not withdraw their hand, but resisted the full train of shocks. The topography and peak amplitudes for the *no go* conditions are shown in Fig. 6 and match that of shock 1 (see also highlighted section *B* in Fig. 3). A paired *t* test comparison revealed a significant difference between the two *no go* conditions, with the internal *no go* again showing a stronger potential in response to itchy shock compared to external *no go* (*t*_14_ = −2.33, *p* = 0.03).

To compare the neural response to shocks 1 and 2, a 2 × 2 ANOVA of the *no go* responses with the factors *shock* and *source of decision* was carried out. Results showed no main effect of shock (*F*_1,14_ = 1.202, *p* = 0.29), a main effect of decision source (*F*_1,14_ = 7.114, *p* = 0.01), with internal *no go* trials showing stronger ERP than external *no go* trials. There was no interaction effect (*F*_1,14_ = 0.21, *p* = 0.65).

Shock 3 was not analysed because participants reported at debriefing that they rarely felt it. This reflected a strong habituation, and the evoked potentials were correspondingly weak.

## Discussion

A paradigm that generates strong urges to make hand withdrawal movements was used to study the inhibitory functions involved in self-control over one’s own actions. Further, situations of internally and externally triggered inhibition were compared within the same context. Participants were either instructed, or decided for themselves, whether to withdraw their right hand from a response pad, thus terminating a train of unpleasantly itchy shocks delivered via a customized electrode to the left forearm.

This paradigm therefore allowed us to study the consequences of inhibition, following both internal decisions and external signals. Specifically, we investigated how internal inhibition influenced neural processing of subsequent itchy shocks, in comparison to externally instructed inhibition. In both these conditions, there is no overt behaviour. However, the source of the decision to inhibit differs between these conditions. We show that the source of inhibitory decisions has an important structuring effect on subsequent sensory experience. This is, to our knowledge, the first study to address the consequences of self-control and internal inhibition of action on somatosensory processing.

### Ecological validity

Our experiment used aversive stimulation to induce a strong urge to act. Unlike previous studies of internal inhibition, we therefore made the choice between action and inhibition motivationally significant. We combined this with the conventional comparison between externally instructed and internally decided actions (Lau et al. 2004), in which participants are asked to ‘freely choose’ between two alternatives roughly half the time. The motivational element of the experiment may seem to conflict with the free selection element: if itchy shocks were truly aversive, participants should choose to avoid them. Our participants’ willingness to accept the experimental instructions perhaps lead them to trade shocks for money (Talmi et al. 2009). However, the balance of positive and negative affect indubitably plays a major role in shaping everyday action choices (Damasio and Dolan 1999). Notwithstanding these general motivations, our participants made a fresh choice on every internally decided trial, whether to act or inhibit *on that trial*. The random intermingling of internally decided and externally instructed trials was designed to discourage them from preparing sequences of ‘free’ choices extending over several trials. For these reasons, our study may have an ecological validity lacking in previous studies of internal inhibition.

### Neural responses to the electrical shocks

A marked positive-going component occurs in response to each of the three shocks, peaking at around 400 ms after shock onset. Previous studies using similar stimuli (Mochizuki et al. 2009) had reported slower positive components, peaking at around 900 ms. We can only speculate on the sources of this difference. Mochizuki et al. associated the long latency of the evoked response with C-fibre activation, because of their slow conduction speed. Indeed, itch sensation has been mainly associated with C-fibres (Handwerker 2010). However, some Aδ-fibres have also been associated with itch in monkeys (Schepers et al. 2008) and humans (Ringkamp et al. 2011). Thus, we speculate that our pulses may also have stimulated both C-fibres and the faster-conducting Aδ-fibres. Activation of Aδ-fibre can lead to both sharp and burning pain. The joint activation of different fibre populations may have produced the distinct sharp itchy feeling that our participants experienced.

In our paradigm, participants always received the first shock and received the second and third shocks only if they chose to inhibit the action of withdrawing their arm. The third shock was perceived very weakly and accordingly produced only a small ERP. Previous reports (Mochizuki et al. 2009) suggested that diminishing neural activity evoked by itch reflected habituation effects. Decrease in saliency for repeated stimuli has been widely reported (Legrain et al. 2011).

*Go* and *no go* trials cannot easily be compared directly, because several different factors may contribute to the differences between their neural correlates. Crucially, *go* trials involve motor preparation, whilst *no go* trials do not. We therefore do not draw any strong conclusions about differences between *go* and *no go* trials. However, the comparing internal and external sources of action decisions is possible, within both the *go* and *no go* condition, because the motor activity is balanced between the two sources of decision.

The neural response for shock 1 produced lower ERP amplitudes when participants internally decided to withdraw their hands, as opposed to when they were externally instructed to do so. The neural response to shock 2 cannot be evaluated in the case of movement conditions, because when the hand has been withdrawn, no further shocks are delivered. However, in the case of *no go* trials, the neural trace of an aversive stimulus that could have been avoided, but was not, could be informative about the mechanisms of inhibition. In these trials, the response to the first and second shock can be evaluated. The neural response to both the first and second shocks was significantly *larger* when participants underwent the shocks as a result of their own internal decision, rather than as a result of an external instruction.

Thus, whilst action trials showed *smaller* ERPs to itchy stimuli in the internally decided *go* trials than in externally instructed *go* trials, inhibition trials showed the opposite effect. We found *stronger* ERPs for internally decided *no go* trials as compared with externally instructed *no go* trials. We speculate that this interaction effect reflects differences in allocation of attention strategies between *go* and *no go* trials. In our paradigm, participants presumably attended to the shocks when they needed to react quickly (*i*.*e*. in *go* conditions). In *no go* trials, the shock is an aversive but inevitable stimulus that is therefore not attended. In *no go* conditions, participants may have preferred to ‘think of something else’ and try to completely ignore the shocks. Interestingly, in internally decided trials, this effect is reversed. That is, the attention allocation towards the aversive stimuli in *go* trials and away from aversive stimuli in *no go* trials would have been less efficient in the internal conditions, according to this view. It has been suggested (Fleming et al. 2009) that internal decisions for actions are less definitive, and easier to change, than externally instructed actions. Could this explain the differences we found between the processing of aversive stimuli in internal and external *no go* trials? If Fleming et al. are correct that internal decisions to act or inhibit still leave open the counterfactual possibility, we speculate that consequences of internal decisions might be strongly processed because of feelings of regret for the missed opportunity of doing otherwise.

In other words, external trials have clear instruction, and there is a clear correct answer. This is not the case with internal trials, in which any course of action would be correct. Therefore, in line with results reported by Fleming et al. (2009), attention allocation in internal trials may represent an ‘intermediate’ situation between the two extremes: allocation of attention towards an aversive stimulus in external *go* trials and allocation of attention away from an aversive stimulus in external *no go* trials.

An alternative, but closely related interpretation, relates to motor processing. In this study, we aimed at investigating action inhibition *indirectly* by addressing the sensory processing of its consequences. However, motor and sensory processes were not temporally segregated in our task. Moreover, EEG techniques do not allow us to unequivocally identify the sources of the modulation of the shock components as either clearly sensory or clearly motor. Thus, it remains possible that our results reflect movement-related processing. Because we did not investigate the periods before action directly, or EEG components that are classically related to action, the hypothesis that our peak ERP amplitude is affected by motor processes remains speculative. The influence of motor preparation on the shock component amplitude is unclear. Importantly, however, this interpretation remains compatible with the ‘intermediate’ account of internal decisions that we suggest above.

Intermediate ERP peak amplitudes may therefore reflect intermediate levels of motor preparation. In particular, whereas external *go* trials are associated with a high levels of motor preparation, internal *go* trials seem to present lower levels of motor preparation, closer to the *no go* conditions. In turn, external *no go* trials are presumably associated with lower levels of motor preparation because no action should occur in *no go* trials. Internal *no go* trials present higher levels of action preparation, closer to *go* trials.

Crucially, in line with the results reported by Fleming et al. (2009), we suggest that internal decisions for action represent situations that are *less* committed to than instructed decisions, and therefore may be easier to change than corresponding external decisions. If the attention allocation account is correct, our results may interestingly extend this interpretation from the purely motor processing addressed by Fleming et al. to the sensory processing of decision consequences.

Inhibition of action can take two rather different forms depending on its time-course. First, it can be a rather tonic behavioural control, related to the concept of willpower (Baumeister et al. 2007; Vohs and Schmeichel 2003). For example, someone who exerts self-control over their eating behaviour may need to continuously inhibit the urge to eat. These inhibitory processes are continuous and ongoing, rather than discrete and precisely timed. Inhibitory self-control may also appear in a more phasic form, as a last-minute inhibition of specific and discrete action impulses (i.e. veto) (Libet 1999). The type of inhibition required in our task lies somewhere on the continuum between these two extremes. Because some trials required inhibition and others required a quick action, the task was designed to encourage phasic, discrete inhibition, rather than generalized, tonic self-control. The present results therefore address the concept of self-control or ‘willpower’ in a novel experimental way.

Here, by focusing on the *consequences* of internal inhibition on subsequent sensory processing, we have provided an event-related measure of internal inhibition, suitable for comparison with instructed inhibition.

## Conclusion

Everyone recognizes the agonizing situation of wanting to respond to an aversive stimulus, but forcing oneself not to. For example, we know that we should not scratch an itch mosquito bite, or drop a cup of coffee that feels too hot. In such cases, we internally inhibit a prepotent action. Our results support the concept of a specific process of internal inhibition, by showing the strong structuring effect of voluntarily withholding action on subsequent sensory processing. When participants internally inhibited an action which would have spared them from itchy shocks, the neural response to those shocks was stronger than when the same choice was made externally. The consequence of a decision to inhibit seems to be more strongly processed than the consequence of an instructed inhibition. When we are given an external instruction, no other action choice is available, and it is relatively easy to grin and bear an aversive stimulus. In contrast, when we ourselves decide what to do, the sensory consequences of our decisions are experienced all the more strongly. This may be because we could have avoided these consequences by choosing otherwise.
